# Factors affecting communication time in an emergency medical communication centers

**DOI:** 10.1186/s13049-024-01315-w

**Published:** 2025-01-13

**Authors:** Melisande Bensoussan, Mathilde Vannier, Thomas Loeb, Jérémie Boutet, Frédéric Lapostolle, Paul-Georges Reuter

**Affiliations:** 1 Samu des Hauts-de-Seine, Assistance Publique-Hôpitaux de Paris, Hôpital Raymond Poincaré, Garches, 92380 France; 2 SAMU 93 - UF Recherche-Enseignement-Qualité, Université Paris 13, Assistance Publique-Hôpitaux de Paris, Sorbonne Paris Cité, Inserm U942, Hôpital Avicenne, 125, rue de Stalingrad, Bobigny, 93009 France; 3 Service des Urgences, SAMU, SMUR, CHU Pontchaillou, Université Rennes, Rennes, France; 4 Univ Rennes, EHESP, Arènes UMR 6051, RSMS U1309, Rennes, France; 5 Pontchaillou Hospital, Rennes, France

## Abstract

**Background:**

Emergency Medical Communication Centres (EMCCs) play a crucial role in emergency care by ensuring timely responses through telephone triage. However, extended communication times can impede accessibility, patient triage, and decision-making. Identifying the factors influencing communication duration is essential for improving EMCC efficiency.

**Objective:**

This study aims to identify temporal, human, and contextual factors associated with prolonged communication times in an EMCC where decision-making is conducted by physicians.

**Methods:**

We conducted a retrospective observational study of all calls received at a French EMCC between March 1 and December 31, 2019. A total of 108,548 patient medical files were analyzed, excluding calls from medical personnel or hospitals. We examined the total communication time (from call initiation to decision) and the medical communication time (physician involvement). Bivariate and multivariate logistic regressions were used to identify factors associated with prolonged communication times.

**Results:**

The median total communication time was 7 min [IQR 5–11], and the median medical communication time was 3 min [IQR 2–4]. Psychiatric reasons for calling (OR = 1.75) and elderly patients (OR = 1.58) were associated with longer communication times. Calls leading to medical advice (OR = 1.48) and calls during weekends or nighttime were also significant factors. Conversely, calls for trauma or from nursing homes, and those handled by emergency physicians, were associated with shorter durations.

**Conclusion:**

Several factors influence communication times in EMCCs, including patient demographics, reason for the call, and time of day.

## Introduction

The structure of Emergency Medical Communication Centres (EMCCs) varies widely. They share a common characteristic: they provide a telephone response as a first line of contact. However, this organization faces several challenges. The first challenge is the increasing volume of calls received. The second challenge is administrative, particularly regarding the precise location of patients. The third is patient triage, which is essential for quickly identifying the most critical patients, such as cardiac arrest victims. Timely identification facilitates the rapid implementation of resuscitation maneuvers and the dispatch of appropriate emergency services. Other patient categories requiring early detection include those with, or at risk of, acute coronary syndrome, acute respiratory distress, stroke, or severe trauma. Depending on the organizational model, their management may begin in the prehospital setting. Identify these patients implies to answer to their call quickly, which is complicated amid the substantial flow of incoming calls. Longer call times reduce the population’s accessibility to EMCC [1]. Furthermore, longer call times could increase 1- and 30- day mortality in aged population [2].

It is crucial to identify factors that contribute to delays between the initiation of a call and decision-making. Language barriers, for instance, have been shown to increase the time to dispatch basic life support teams by 33% and acute life support teams by 43% [3]. Such difficulties have been reported in over a third of cases [4]. Factors such as language barriers, critical situations, or psychiatric reasons for calling complicate communication and delay decision-making [5]. Reducing decision-making delays can positively impact patient outcomes and is also a significant organizational goal.

In France, the EMCC operates on a two-tiered system [6]. Calls are initially handled by call takers who have completed a one-year degree course. They are responsible for answering the call, filling out the patient’s medical file (PMF), locating the patient, identifying the reason for the call, and assessing the urgency level. Based on this prioritization, calls are directed either to an emergency physician or a general practitioner. This study aims to identify the factors associated with longer communication times in a system where regulation and decision-making are conducted by a physician.

## Methods

### Design

We conducted an observational, retrospective, single-center study from March 1, 2019, to December 31, 2019, concerning all calls received over this period. The year 2019 was chosen to reflect the situation without the impact of the SARS-CoV-2 pandemic. The analysis began in March due to an update of the regulation software.

### Setting

France is divided into 13 regions and 100 departments. With a few rare exceptions, each department has its own EMCC. The EMCC of the “Hauts de Seine” department (EMCC 92) is located in Garches, at the University Hospital Raymond-Poincaré, in the inner suburbs of Paris. It covers an area of 176 km^2^ with a population of 1.6 million.

### Population

We included all calls originating from non-medical personnel and outside a hospital structure. Criteria for non-inclusion were calls for inter-hospital transfer and calls from community-based physicians.

### Extracted data

To ensure the completeness of the data, the variables collected were those standardized in the medical regulation software Centaure^®^.

- The time factors were date, day, and call time. A school holiday variable was created. A daytime hour was defined between 8:00 am and 7:59 pm, and a nighttime hour between 8:00 pm and 7:59 am. A working hour was defined as a daytime hour on a weekday, excluding school holidays. Non-working hour included nighttime hours, weekends, and school holidays.
- The reasons for the call, as coded by the call taker, were collected: cardiological (including cardiac arrest), traumatological, respiratory, neurological, death, other medical reasons (allergy, diabetes, abdominal pain, fever, gyneco-obstetrics, hemorrhage, ENT (ear, nose and throat)/stomatology/ophthalmology), toxicology or neonatology/pediatrics. The caller’s initial request was notified as a request for a doctor at home, medical advice, a call for help, a request for an ambulance and other requests (other health professionals, looking for a place). The place of intervention could be a home, workplace, school, public place, retirement home, doctor’s office, or other (prison, police station). The caller category was collected: the subject himself, a third party (including family or from another call center), or a paramedic.
- Age and sex of the patient were collected.
- The time of medical regulation and the type of doctor (emergency doctor or general practitioner) were recorded. The time and type of medical decision were recorded: dispatch of rescue team (ambulance, first-aider, fire brigade, mobile intensive care unit), advice (medical advice, contact details of a pharmacy on call) or permanent care physician (consultation at the medical center, dispatch of a doctor from the home emergency medical service), as well as the profile of the person who triggered it: physician or non-medical staff.

### Objective

The aim was to determine the temporal, human and contextual factors influencing total communication time during an EMCC call.

The primary endpoint, total communication time, was defined as the delay between picking up the phone at the EMCC and making a decision. The secondary endpoint, medical communication time, was defined as the time between the start of the physician’s notetaking and the decision being made.

### Statistical analysis

The results are presented in accordance with the RECORD recommendations for observational studies, inspired by the STROBE recommendations. The analysis was based on complete data. No data imputation was performed. Quantitative data are expressed as mean and standard deviation or median and interquartile range, depending on their distribution. Categorical variables are presented as numbers and percentages.

Logistic regressions were performed to identify factors associated with total communication time. The judgment criterion was dichotomized at its median. To account for inter-doctor variability, and given an inter-class correlation coefficient of 12%, we used mixed models with the EMCC physician at level 2. The multivariate analysis was carried out using a mixed logistic regression model, with the attending physician at level 2. The variables included in the model were those associated with a *p* < 0.2 in bivariate analysis. In cases where several variables reflected the same data (e.g. temporality with the “working hour” variable), several models were tested. The variables selected for the multivariate model were those with the lowest Akaike information criterion. For secondary endpoints, variables were compared by Student’s or Mann-Whitney’s T-test for quantitative variables according to their distribution, and by Fisher’s test or Chi2 for qualitative variables. The significance threshold was set at 5%. Analyses were performed using R software (version 4.2.0).

## Results

Over the period, EMCC 92 created 149,167 PMFs. After applying the inclusion and non-inclusion criteria, 108,548 PMFs were retained for analysis.

### Descriptive analysis

Medical regulation files were created during working hours in 76,726 (71%) cases. The population comprised 60,745 (58%) women with a median age of 40 [IIQ: 22–70] years and 45,227 (42%) men with a median age of 35 [IIQ: 8–62] years. The global median age was 38 [IIQ: 18–66] years. The caller was a third party for 69,526 (64%) calls. The patient was at home in 91,481 (84%) cases. The three most frequent reasons for calling were cardiological (37%), neonatal/pediatric (29%) and other medical (19%). The call taker referred 85,701 (79%) calls to general practice. The medical decision was medical advice for 52,894 (49%) and the dispatch of rescue team for 41,100 (38%) calls. The median time from file creation to decision was 7 [IIQ: 5–11] minutes. The median medical communication time was 3 [IIQ: 2–4] minutes. The results of the descriptive analysis are presented in Table 1.

**Table 1:** Descriptive and bivariate analyses of variables depending on the communication time

	Total *n*=108 548	Time below median *n*=55 551 (51%)	Time above median *n*=52 997 (49%)	*P*
**Temporal variables**				
**Week / week-end**				
Week	74 526 (69%)	38 870 (70%)	35 556 (67%)	< 0.001
Weekend	34 122 (31%)	16 681 (30%)	17,441 (33%)	
**Non-Holiday/holiday**				
Non-holiday	104 243 (96%)	53 210 (96%)	50 993 (96%)	< 0.001
Holiday	4 345 (4%)	2341 (4%)	2004 (4%)	
**Day / Night**				
Day	61 699 (57%)	32 278 (58%)	29 421 (56%)	< 0.057
Night	46 849 (43%)	23 273 (42%)	23 576 (44%)	
**Schedule**				
Non-working hours	31 822 (29%)	17 443 (31%)	14 379 (27%)	< 0.001
Working	76 726 (71%)	38 108 (69%)	38 618 (73%)	
**Patients variables**				
Sex*				
Female	60 745 (58%)	30 750 (57%)	30 045 (58%)	< 0.01
Male	45 227 (42%)	23 365 (43%)	21 862 (42%)	
Age**				
[0 ; 18)	26 782 (25%)	14 812 (27%)	11 970 (23%)	
[18 ; 38)	26 675 (25%)	13 625 (25%)	13 050 (25%)	< 0.001
[38 ; 66)	27 199 (25%)	13 391 (24%)	13 808 (26%)	< 0.001
[66 ; 109]	27 391 (25%)	13 449 (24%)	13 942 (26%)	< 0.001
**Contextual variables**				
**Reasons**				
Cardiological	40 2454 (37%)	19 785 (36%)	20 496 (39%)	
Others	30 968 (29%)	15 498 (28%)	15 471 (29%)	< 0.001
Medical other	20 597 (19%)	10 843 (19%)	9 754 (18%)	< 0.001
Trauma	8 791 (8%)	5 177 (9%)	3 614 (7%)	< 0.001
Respiratory	3 916 (4%)	2 254 (4%)	1 662 (3%)	< 0.001
Neurological	1 584 (2%)	896 (2%)	688 (1%)	< 0.001
Psychiatric	1 474 (1%)	552 (1%)	922 (2%)	< 0.001
Intoxication	710 (<1%)	410 (< 1%)	300 (<1%)	< 0.001
Deceased	253 (<1%)	136 (< 1%)	117 (<1%)	< 0.016
**Requests**				
Physician	47 686 (44%)	23 718 (43%)	23 968 (45%)	
Medical Advice	38 720 (36%)	19 844 (36%)	18 876 (37%)	< 0.001
Call for help	13 572 (11%)	7 669 (14%)	5 903 (11%)	< 0.001
Ambulance	8 342 (8%)	4 214 (7%)	4 128 (7%)	0.9
Other request	228 (1%)	106 (<1%)	122 (<1%)	0.4
**Intervention locations**				
Home	91 481 (84%)	46 313 (83%)	45 168 (85%)	
Workplace	5 457 (5%)	2 618 (5%)	2 839 (5%)	< 0.001
Public place	5 032 (5%)	2 828 (5%)	2 204 (4%)	< 0.001
Retirement home	3 618 (3%)	2 062 (4%)	1 556 (3%)	< 0.001
School	2 346 (2%)	1 420 (3%)	926 (2%)	< 0.001
Other	406 (<1%)	187 (<1%)	219 (<1%)	0.013
Doctor’s office	208 (<1%)	123 (<1%)	85 (<1%)	0.057
**Caller**				
Subject	33 493 (31%)	20 062 (36%)	20 181 (38%)	
Third party	69 526 (64%)	32 556 (59%)	30 223 (57%)	< 0.001
Paramedical	5 526 (5%)	2 933 (5%)	2 593 (5%)	0.037
**Decision variables**				
**Decision profile**				
General Practitioner	82 489 (76%)	17 123 (31%)	16 370 (31%)	
Emergency Physician	18 744 (17%)	35 495 (64%)	34 034 (64%)	<0.001
Non-medical staff	7 315 (7%)	2 933 (5%)	2 593 (5%)	<0.001
**EMCC Physician**				
General Practitioner	85 701 (79%)	42 122 (76%)	43 579 (82%)	
EMS Physician	22 847 (21%)	13 429 (24%)	9 418 (18%)	0.001
**Decision**				
Rescue dispatch	41 100 (38%)	22 386 (40%)	18 714 (35%)	
Advice	52 894 (49%)	25 785 (46%)	27 109 (51%)	< 0.001
General practitioner	14 554 (13%)	7 380 (13%)	7 174 (14%)	0.2

Results are given in number (percentage)

* 2,526 calls (2.3%) not assigned. ** 501 calls (0.5%) not assigned

### Bivariate analysis

The results of the bivariate analyses are presented in Table 1. All variables were retained for multivariate analysis.

### Multivariate analysis

Multivariate analysis revealed 24 variables associated with communication duration. Eleven variables were significantly associated with increased total communication time: calls for psychiatric reasons, a call involving for patient older than 18 years old, when the decision was medical advice, calls from workplace and other locations, calls at weekends or at night, referral to general practice and calls for an ambulance. Thirteen variables were significantly associated with reduced total communication time: calls for the discovery of a deceased person, traumatological reason, toxicological reason, respiratory reason, neurological reason, medical other and neonatology/pediatrics, calls directed to emergency medicine, calls from a nursing home or from the public highway, calls for medical advice or help and calls on public holidays. The results are presented in Table 2.

**Table 2:** Multivariate analyses of factors associated with a longer communication time

Variables	Odds ratio [IC 95%]	*P*
**Temporal variables**		
**Week / week-end**		
Week	1.00	
Weekend	1.08 [1.05-1.12]	< 0.001
**Non-holiday/holiday**		
Non-holiday	1.00	
Holiday	0.87 [0.81-0.93]	< 0.001
**Day / Night**		
Day	1.00	
Night	1.04 [1.01-1.08]	0.012
**Contextual variables**		
**Reasons**		
Cardiological	1.00	
Others	0.93 [0.90-0.97]	< 0.001
Medical other	0.86 [0.83-0.89]	< 0.001
Trauma	0.69 [0.66-0.73]	< 0.001
Respiratory	0.83 [0.77-0.89]	< 0.001
Neurological	0.88 [0.79-0.99]	0.030
Psychiatric	1.75 [1.56-1.96]	< 0.001
Intoxication	0.78 [0.66-0.91]	0.002
Deceased	0.63 [0.48-0.82]	< 0.001
**Requests**		
Physician	1.00	
Medical Advice	0.93 [0.91-0.96]	< 0.001
Call for help	0.85 [0.81-0.89]	< 0.001
Ambulance	1.08 [1.02-1.14]	0.005
Other request	1.10 [0.82-1.48]	0.5
**Intervention locations**		
Home	1.00	
Workplace	1.26 [1.18-1.34]	< 0.001
Public place	0.92 [0.86-0.98]	0.006
Retirement home	0.75 [0.69-0.81]	< 0.001
School	0.98 [0.89-1.08]	0.7
Other	1.37 [1.10-1.69]	0.004
Doctor’s office	0.76 [0.57-1.03]	0.073
**Decision variables**		
**EMCC Physician**		
General Practitioner	1.00	0.020
EMS Physician	0.71 [0.53-0.95]	
**Decision**		
Rescue dispatch	1.00	
Advice	1.48 [1.43-1.52]	< 0.001
General practitioner	1.08 [1.04-1.13]	< 0.001
**Patients variables**		
Sex*		
Female	1.00	
Male	0.98	0.10
Age**		
[0 ; 18)	1.00	
[18 ; 38)	1.19 [1.14-1.24]	< 0.001
[38 ; 66)	1.33 [1.28-1.39]	< 0.001
[66 ; 109]	1.58 [1.52-1.65]	< 0.001

## Discussion

Communication times between the call taker, the EMCC physician, and the caller varied significantly. 25% of calls lasted less than 5 min, while another 25% exceeded 11 min. Overall, communication time was evenly divided among call-taking, administrative data collection, and severity assessment by the caller, as well as the purely medical aspect. Several factors influenced these communication times, including psychiatric reasons for calling (OR = 1.75) and the elderly (OR = 1.58) (Table 2). The nature of the medical decision also played a crucial role, with the provision of medical advice notably extending communication time (OR = 1.48) (Table 2).

Reducing communication times poses a challenge for EMCCs globally. Such reductions benefit not only patients but also call takers, EMCC physicians, and the healthcare system. For patients, quicker decision-making can lead to faster dispatch of emergency services in distress situations. For call takers and regulating physician, reducing time spent on calls can alleviate workload. For healthcare system, reducing response time can enhance accessibility for patients [1]. Improved accessibility would contribute to better quality of care, resulting in greater patient satisfaction and reduced staff burnout. The introduction of a two-level call answering system in France is one approach to enhancing accessibility [7–9].

The main factors identified in our study as contributing to longer communication times align with those previously reported as generating communication difficulties [3–5, 10]. Therefore, optimizing communication is key to reducing call duration, necessitating dedicated training for call takers and regulating physicians. In France, this training is now structured and incorporates theoretical and simulation-based components. Although limited in number, emerging studies indicate the positive impact of specific training on caregiver communication skills. A recent randomized study demonstrated that dedicated coaching significantly improved physicians’ levels of empathy [11]. This is particularly important, as enhanced communication can help mitigate the risk of post-traumatic stress disorder, which has been reported in over a quarter of call takers [12].

In the French EMCC system, calls are managed by a physician who may be either a general practitioner or an emergency physician, depending on the call’s nature. The median medical communication time was 3 [2–4] minutes. These results are consistent with the literature, which reports that an emergency physician makes a diagnostic hypothesis in less than 5 min [13]. It follows that calls handled by emergency physicians are generally associated with shorter communication times, as decisions can often be made swiftly in cases of suspected cardiac arrest or obvious distress. Conversely, communication times lengthen when calls conclude with the provision of medical advice. However, this strategy ultimately benefits the healthcare system by preventing unnecessary medical consultations or emergency room visits. Previous research has shown that structured telephone dispensing of medical advice can significantly reduce rates of GP consultations and emergency room visits for common complaints such as fever or gastroenteritis [14]. High patient satisfaction (90%) and substantial savings (€91 vs. €150; *p* < 0.01) further support the efficacy of this approach. The flow of calls related to non-urgent issues (79%), often resulting in extended telephone medical advice, contributes to increased waiting times between the call taker and the regulating physician, likely without impacting patient morbidity in this population.

Finally, new tools are being developed to optimize and expedite decision-making. For example, chest pain is a frequent reason for calls to EMCCs, yet satisfactory decision-making algorithms are lacking. The integration of dedicated software and artificial intelligence has the potential to address this gap. While some studies have focused on cardiac arrest, results have not yet met expectations [15, 16]. Conditions such as cardiac arrest, myocardial infarction, stroke, and respiratory distress are critically time-sensitive, making prompt recognition essential. Additionally, the implementation of specialized channels, such as the inclusion of nurses specializing in psychiatry or geriatrics, is currently under evaluation.

These results must be interpreted considering several limitations. Firstly, as previously mentioned, the organization of the French EMCC is specific. Secondly, our population was younger than in other studies. In Copenhagen, individuals aged over 66 represented 44% of the population [17], while in our study, they accounted for 25%. Thirdly, we defined our outcomes as the time when the medical decision was recorded in the software. This time may be overestimated, as the physician might provide an explanation before entering the decision into the system. Lastly, we were unable to collect data on the availability of call takers at specific times or on the workload during EMCC operations. Similarly, we had no information on the availability of rescue resources during calls, nor on any modification of decisions by call takers in the event of resource unavailability. These regulatory factors could be confounding and may influence our results.

## Conclusion

Numerous factors influence communication times in emergency medical communication centers (EMCCs). Identifying these factors will guide the organization of responses within EMCCs.

## Data Availability

The statistical code and technical processes are available from the time of publication. Appropriate institutional agreements will be required for anonymized participant data transfer. Requests should be made via email to the corresponding author along with an analysis proposal.

## Declarations

### Transparency declaration

The lead authors (the manuscript guarantors) affirm that the manuscript is an honest, accurate, and transparent account of the study being reported; that no important aspects of the study have been omitted; and that any discrepancies from the study as planned (and, if relevant, registered) have been explained.
