# Correction: Factors affecting communication time in an emergency medical communication centers

**DOI:** 10.1186/s13049-025-01356-9

**Published:** 2025-03-04

**Authors:** Melisande Bensoussan, Mathilde Vannier, Thomas Loeb, Jérémie Boutet, Frédéric Lapostolle, Paul-Georges Reuter

**Affiliations:** 1 Samu des Hauts-de-Seine, Assistance Publique-Hôpitaux de Paris, Hôpital Raymond Poincaré, Garches, 92380 France; 2 SAMU 93 - UF Recherche-Enseignement-Qualité, Université Paris 13, Assistance Publique-Hôpitaux de Paris, Sorbonne Paris Cité, Inserm U942, Hôpital Avicenne, 125, rue de Stalingrad, Bobigny, 93009 France; 3 Service des Urgences, SAMU, SMUR, CHU Pontchaillou, Université Rennes, Rennes, France; 4 Univ Rennes, EHESP, Arènes UMR 6051, RSMS U1309, Rennes, France; 5 Pontchaillou Hospital, Rennes, France

**Correction to: Bensoussan et al. Scandinavian Journal of Trauma, Resuscitation and Emergency Medicine (2025) 33:6**

10.1186/s13049-024-01315-w.

Following the publication of the original article, the authors reported that the name of the 2nd author was misspelled.

**Incorrect**

Mathilde Vanier

**Correct**

Mathilde Vannier

This correction shows the correct name for all authors. The original article has been corrected.

